# Inhibition of Immune Complex Complement Activation and Neutrophil Extracellular Trap Formation by Peptide Inhibitor of Complement C1

**DOI:** 10.3389/fimmu.2018.00558

**Published:** 2018-03-26

**Authors:** Pamela S. Hair, Adrianne I. Enos, Neel K. Krishna, Kenji M. Cunnion

**Affiliations:** ^1^Department of Pediatrics, Eastern Virginia Medical School, Norfolk, VA, United States; ^2^Children’s Specialty Group, Norfolk, VA, United States; ^3^Department of Microbiology and Molecular Cell Biology, Eastern Virginia Medical School, Norfolk, VA, United States; ^4^Children’s Hospital of The King’s Daughters, Norfolk, VA, United States

**Keywords:** immune complex, complement system, neutrophil extracellular traps, NETosis, myeloperoxidase, peptide inhibitor of complement C1

## Abstract

Two major aspects of systemic lupus erythematosus (SLE) pathogenesis that have yet to be targeted therapeutically are immune complex-initiated complement activation and neutrophil extracellular trap (NET) formation by neutrophils. Here, we report *in vitro* testing of peptide inhibitor of complement C1 (PIC1) in assays of immune complex-mediated complement activation in human sera and assays for NET formation by human neutrophils. The lead PIC1 derivative, PA-dPEG24, was able to dose-dependently inhibit complement activation initiated by multiple types of immune complexes (IC), including C1-anti-C1q IC, limiting the generation of pro-inflammatory complement effectors, including C5a and membrane attack complex (sC5b-9). In several instances, PA-dPEG24 achieved complete inhibition with complement effector levels equivalent to background. PA-dPEG24 was also able to dose-dependently inhibit NET formation by human neutrophils stimulated by PMA, MPO, or immune complex activated human sera. In several instances PA-dPEG24 achieved complete inhibition with NETosis with quantitation equivalent to background levels. These results suggest that PA-dPEG24 inhibition of NETs occurs by blocking the MPO pathway of NET formation. Together these results demonstrate that PA-dPEG24 can inhibit immune complex activation of the complement system and NET formation. This provides proof of concept that peptides can potentially be developed to inhibit these two important contributors to rheumatologic pathology that are currently untargeted by available therapies.

## Introduction

The pathogenesis of systemic lupus erythematosus (SLE) is very complex, but two major contributors are immune complex-initiated complement activation and neutrophil extracellular trap (NET) formation. Immune complexes (IC) initiating classical pathway complement activation leading to consumption of C4 and C3 have long been appreciated and clearly contribute to lupus nephritis (1–3). NETs, however, are more recently recognized as contributing to SLE pathogenesis, with convincing evidence accruing rapidly in recent years (4–6).

The role of anti-C1q antibodies in the blood of SLE patients is an active area of investigation with considerable data accumulating to demonstrate a strong association between the presence of anti-C1q antibodies and lupus nephritis (7, 8). Investigators have also shown that anti-C1q antibodies from SLE patients bound to a surface in an ELISA-type assay can activate the classical and lectin pathways (9). Thus, anti-C1q antibodies appear to be important in pathogenesis and deserve consideration when modeling SLE-like IC.

It is known that IC can activate complement generating complement effectors (e.g., C5a, sublytic concentrations of membrane attack complex, etc.) that interact with and can stimulate human neutrophils (10–13). However, articles describing that IC can induce neutrophils to generate NETs have focused on the role of Fc receptors in this process (14–17). These data demonstrate a link between IC and NETs, but to our knowledge the contribution of complement activation in this process remains unexplored. The reverse scenario, NETosing neutrophils activating complement, has previously been reported by Yuen et al (18).

An article by Akong-Moore et al. (19) suggested that a major pathway of NET formation can be mediated by myeloperoxidase (MPO) *via* its primary function of generating hypochlorous acid from hydrogen peroxide (H_2_O_2_) and chloride ion. Another article by Kirchner et al. showed that NADPH oxidase and MPO-derived reactive oxygen species are critical for the formation of NETs (20). Studies reported by Parker et al. demonstrated that PMA-stimulated NET formation could be inhibited with ABAH, an MPO inhibitor (21, 22). These findings suggest that it might be possible to block NET formation by utilizing other MPO inhibitors.

Peptide inhibitor of complement C1 (PIC1) is a family of more than 70 peptides of related sequences that inhibit the classical pathway of complement by binding and blocking activation of the initiating component of the cascade, C1 (23, 24). The original viral-derived peptides have undergone substantial rational drug design to optimize classical pathway complement inhibition and increase solubility, yielding PA-dPEG24. The lead compound, PA-dPEG24, is a 15 amino acid PEGylated molecule that has been shown to inhibit antibody-initiated complement-mediated hemolysis *in vitro* and *in vivo* (25, 26). We have recently shown that PA-dPEG24 can inhibit the peroxidase activity of MPO in sputum from cystic fibrosis patients (27). We have subsequently shown that PA-dPEG24 can inhibit the peroxidase activity of other heme-based molecules *via* interaction with the heme ring (28). In this manuscript, we explore the extent to which PA-dPEG24 can inhibit immune complex-initiated complement activation as well as inhibit NET formation.

## Materials and Methods

### Ethics Statement

Blood from healthy donors was obtained with written informed consent under an Eastern Virginia Medical School IRB approved protocol, 02-06-EX 0216.

### Reagents

PA-dPEG24 (IALILEPICCQERAA-dPEG24) was manufactured by PolyPeptide Group (San Diego, CA, USA) to ≥95% purity verified by HPLC and mass spectrometry analysis. Lyophilized PA-dPEG24 was solubilized in normal saline with 0.01 M Na_2_HPO_4_ buffer to 37.5 mM. Purified MPO was purchased from Lee Biosolutions (Maryland Heights, MO, USA). Intravenous immune globulin was purchased from Baxter Healthcare Corporation (Westlake Village, CA, USA), ovalbumin from Sigma Aldrich (St. Louis, MO, USA), and the antiovalbumin antibody from Abcam (Cambridge, MA, USA). Goat anti-C1q and human C1 were purchased from Complement Technology (Tyler TX, USA). PMA (Phorbol 12-myristate 13-acetate) and H_2_O_2_ were purchased from Fisher Scientific (Waltham, MA, USA). Oxidized PA-dPEG24 was prepared by adding H_2_O_2_ at a final concentration of 0.3% for 10 min at room temperature. Removal of residual H_2_O_2_ was performed by incubating the mixture at 60°C for 1 h.

### Buffers

Complement permissive GVBS^++^ buffer is veronal-buffered saline with 0.1% gelatin, 0.15 mM CaCl_2_, and 1 mM MgCl_2_ (29). Complement inhibitory buffer GVBS^−−^ is a veronal-buffered saline with 0.1% gelatin and 10 mM EDTA.

### Pooled Normal Human Sera (NHS)

Pooled NHS was prepared as previously described (29).

### Immune complex activation of NHS

Normal human sera was stimulated with three different types of IC to induce complement activation as follows. Heat-aggregated IgG (Agg-IgG) was generated by incubating intravenous immune globulin at 50 mg/ml at 63°C for 30 min (30). Ovalbumin–antiovalbumin IC were made by incubating 0.01 ml antiovalbumin antibody with an equal volume of ovalbumin, at 0.25 mg/ml, at 37°C for 30 min, and then storing at 4°C overnight. C1 IC were formed by incubating 0.02 ml anti-C1q goat sera with 5 µl of C1, at 200 µg/ml, at 30°C for 30 min, and then placing in an ice water bath. For C5a and C5b-9 assays, activation of NHS was performed by pre-incubating 5% NHS with titrating concentrations of PA-dPEG24 in 0.3 ml of GVBS^++^ buffer for 30 min at room temperature. Then 2 µl of either heat-aggregated IVIg, or ovalbumin IC, or 5 µl of C1-anti-C1q IC was added to the mix for 30 min at 37°C. This reaction was stopped with the addition of an equal volume of GVBS^−−^. For iC3b detection, 1% NHS was used and the rest of the protocol remained the same.

### ELISAs

Samples were assayed using C5a, iC3b, and SC5b-9 ELISAs. A C5a ELISA kit (R&D Systems) was used per the manufacturer’s instructions. ELISAs for iC3b and SC5b-9 were performed as previously described (31). The iC3b ELISA uses a goat anti-human C3 antibody (Complement Technology, Tyler, TX, USA) for capture, and a mouse anti-human iC3b antibody (Quidel, San Diego, CA, USA) for probing, and a goat anti-mouse HRP antibody for detection. The SC5b-9 ELISA uses a rabbit anti-human SC5b-9 antibody (Complement Technology) for capture, a mouse anti-human SC5b-9 monoclonal antibody (Quidel) for probing, and a chicken anti-mouse HRP antibody for detection. Colorimetric detection was performed with TMB, stopped with H_2_SO_4_, and read on a BioTek Synergy HT plate reader at 450 nm.

### Purified Neutrophils and Neutrophil Lysate

Neutrophils from the blood of healthy volunteers were purified from heparinized blood by Hypaque–Ficoll step gradient centrifugation, dextran sedimentation, and hypotonic lysis, as previously described (32).

### NET Assay in Microtiter Plate

The formation of NETs was induced by incubating 2.0 × 10^5^ neutrophils per well in a 96-well tissue culture plates with RPMI media alone, or adding 0.05% of H_2_O_2_, or 12 nM PMA, or 8 µg/ml MPO, or PA-dPEG24 at various concentrations. For immune complex sera-induced NET formation, activated sera were made by adding 5 µl of ovalbumin–antiovalbumin immune complex to 5% NHS in 0.3 ml of GVBS^++^. This combination was allowed to incubate for 30 min at 37°C, and then 0.05 ml was added to the neutrophils in RPMI. Cells were then incubated for 1.5 h at 37°C in 5% CO_2_ incubator.

### Ellman’s Reagent Assay

Samples were mixed 1:10 into an 80 µg/ml solution of Ellman’s Reagent (Thermo Scientific Pierce, Waltham, MA, USA) diluted in reaction buffer (0.1 M sodium phosphate with 1 mM EDTA, pH 8.0) and allowed to incubate at room temperature for 15 min. A standard curve was generated using cysteine hydrochloride monohydrate (Sigma Aldrich, St. Louis, MO, USA) at concentrations ranging from 0.25 to 1.5 mM in the reaction buffer mixed with the Ellman’s reagent at the same proportion and incubated the same as the samples. Samples and standards were read at an absorbance of 412 nm in a BioTek Synergy HT plate reader and a linear regression was generated and used to analyze the samples yielding a calculation of mM sulfhydryl groups relative to a cysteine standard.

### Quantitation of NET Formation

Free DNA was measured by PicoGreen in the supernatant recovered from the NET microtiter plate well assay (19). Five hundred units of monococcal nuclease (Fisher) were added to each well to allow for digestion of released extracellular DNA for 10 min in 37°C incubator. The preparation was then aliquoted into an adjacent well and mixed 1:1 with prepared PICO green reagent (Fisher). The fluorescence was then quantified on a BioTek microplate reader at excitation 485 nm/emission 528 nm.

### NET Formation for Fluorescence Microscopy

Purified human neutrophils were assayed on a glass slide as follows. Cells were combined with RPMI media and the indicated stimuli as mentioned above in a tube and then aliquoted onto a glass slide circled with a hydrophobic slide marker. The slides were incubated for 1.5 h at 37°C in a 5% CO_2_ incubator at 37° for 1.5 h. Slides were fixed overnight with 4% paraformaldehyde at 4°C. For all staining, the following conditions were used. Slides were washed in PBS and incubated in blocking solution (2% normal goat serum + 2% bovine serum albumin in PBS) for 1 h at room temperature. Then the slides were incubated with primary antibody at 1:300 in 2% BSA in PBS for 1 h at room temperature. Slides were washed in PBS three times and incubated in fluorescent-labeled secondary antibody at 1:1,000 or DAPI (Southern Biotech) at 0.25 pg/ml final in 2% BSA in PBS for 1 h at room temperature. Slides were then washed three times in PBS and were imaged. Cells were visualized using a DP70 Digital Camera (Olympus Center, Valley Forge, PA, USA), mounted on a BX50, Olympus microscope. Staining antibody pairs used were rabbit anti-MPO (Thermo Scientific) and rabbit anti-histone H3 (Abcam) with the secondary goat anti-rabbit Alexa Fluor 488 (Novus Biologicals). Also, mouse anti-elastase (Invitrogen) was used with the secondary goat anti-mouse Alexa Fluor 568 (Novus Biologicals).

### Statistical Analysis

Quantitative data were analyzed determining means, SEM, and Student’s *t*-test (33) using Excel (Microsoft, Redmond, WA, USA).

## Results

### PA-dPEG24 Inhibition of Immune Complex-Initiated Complement Activation

**Figure 1:**
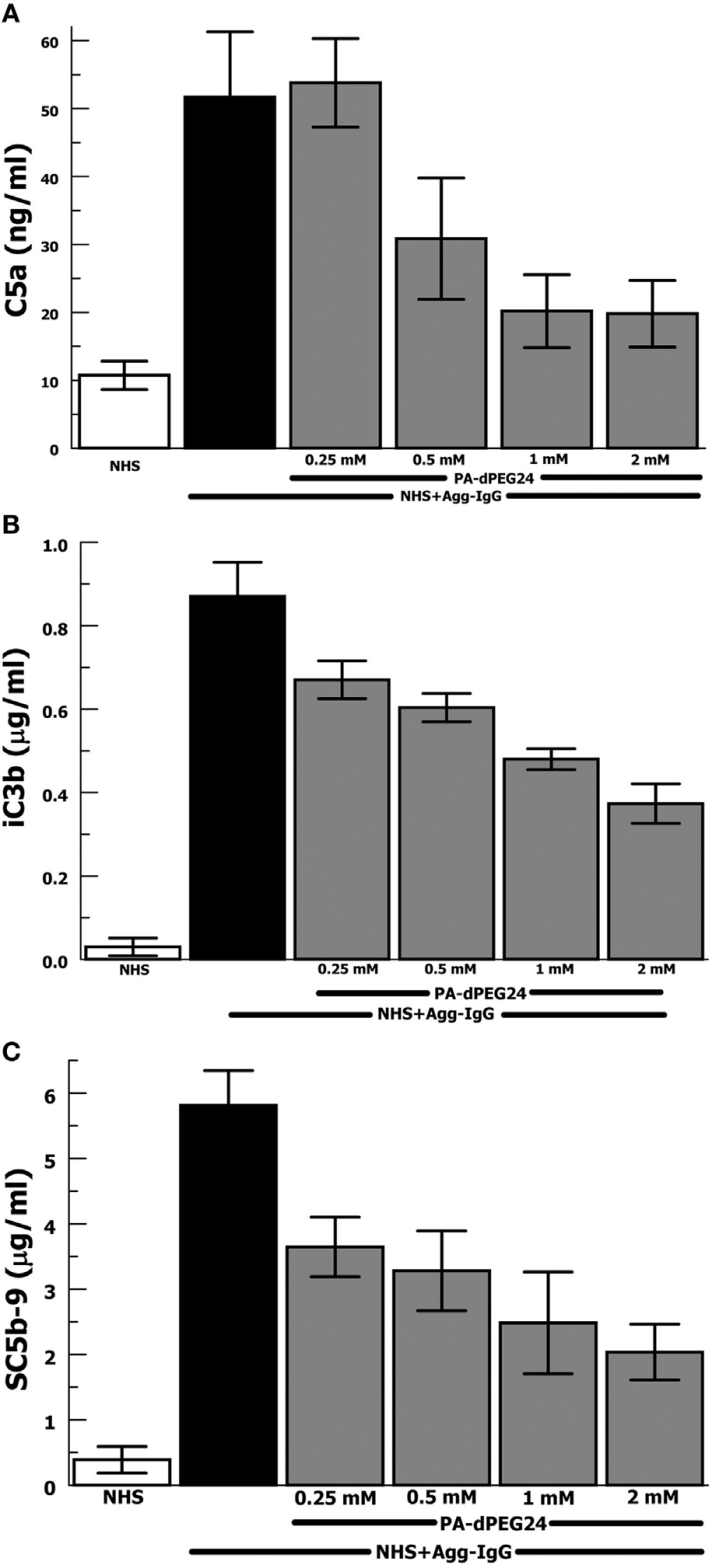
PA-dPEG24 inhibition of heat-aggregated IgG (Agg-IgG) immune complex-initated complement activation assayed by complement effectors. **(A)** PA-dPEG24 inhibition of C5a generation in normal human sera (NHS) stimulated with heat-Agg-IgG immune complexes (IC). Data are means of (*n* = 5) independent experiments ± SEM. **(B)** PA-dPEG24 inhibition of iC3b generation in NHS stimulated with heat-Agg-IgG IC. Data are means of (*n* = 4) independent experiments ± SEM. **(C)** PA-dPEG24 inhibition of SC5b-9 generation in NHS stimulated with heat-Agg-IgG IC. Data are means of (*n* = 6) independent experiments ± SEM.

Our group has previously shown that PA-dPEG24 can inhibit classical pathway-mediated hemolysis in a CH50-type assay (25) as well as inhibit classical pathway-mediated hemolysis *in vitro* and *in vivo* in a mismatched transfusion model (26). In order to evaluate the ability of PA-dPEG24 to inhibit immune complex-initiated complement activation, we utilized the archetypal immune complex stimulant of heat-Agg-IgG (30, 34) in NHS. We assayed for three important effectors resulting from complement activation, the major proinflammatory anaphylatoxin, C5a, a cleavage product of C3 activation, iC3b, and the membrane attack complex, C5b-9 (Figures 1A–C). For each assay, PA-dPEG24 dose-dependently inhibited elaboration of the effector after stimulation with heat-Agg-IgG compared with no inhibitor. Statistically significant inhibition was achieved in each assay at ≥0.5 mM PA-dPEG24 (*p* < 0.05). For C5a, 1 mM PA-dPEG24 lead to a 61% reduction (*p* = 0.002) compared with heat-Agg-IgG with no inhibitor. These results suggest that PA-dPEG24 can inhibit immune complex-initiated complement activation in human sera.

**Figure 2:**
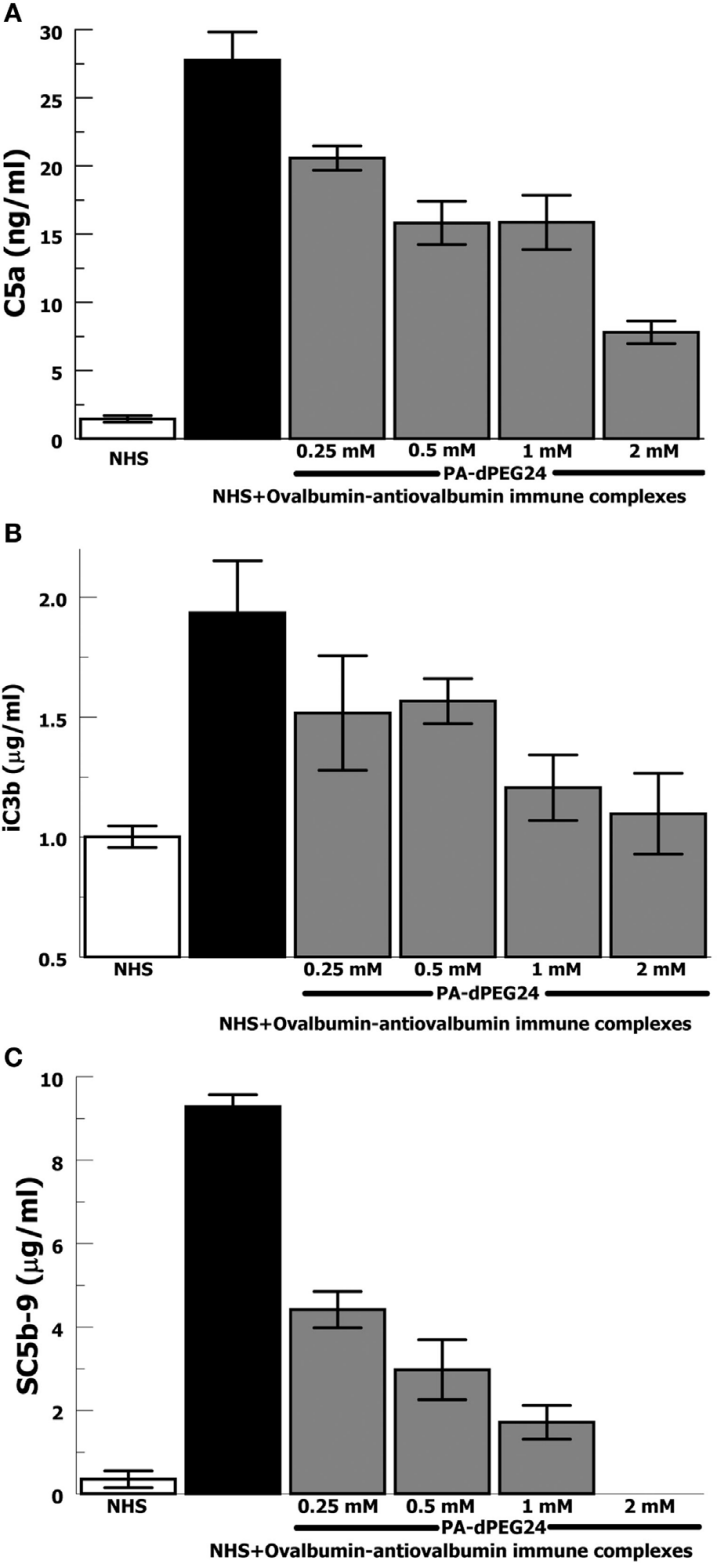
PA-dPEG24 inhibition of ovalbumin–antiovalbumin immune complex-initated complement activation assayed by complement effectors. **(A)** PA-dPEG24 inhibition of C5a generation in normal human sera (NHS) stimulated with ovalbumin–antiovalbumin immune complexes (IC). Data are means of (*n* = 4) independent experiments ± SEM. **(B)** PA-dPEG24 inhibition of iC3b generation in NHS stimulated with ovalbumin–antiovalbumin IC. Data are means of (*n* = 4) independent experiments ± SEM. **(C)** PA-dPEG24 inhibition of SC5b-9 generation in NHS stimulated with ovalbumin–antiovalbumin IC. At 2 mM PA-dPEG24 the measured SC5b-9 was at the lower limit of detection. Data are means of (*n* = 3) independent experiments ± SEM.

In order to provide confirmation of the results with heat-Agg-IgG, we then tested the antigen–antibody immune complex most often utilized in animal models of complement activation, ovalbumin, and antiovalbumin (35, 36). The ovalbumin–antiovalbumin IC were used to stimulate complement activation in NHS and the same three effectors, C5a, iC3b, and C5b-9, were measured. PA-dPEG24 dose dependently inhibited generation of each complement effector with statistically significant inhibition achieved at ≥0.25 mM PA-dPEG24 (*p* < 0.03) compared with ovalbumin–antiovalbumin alone (Figures 2A–C). These results provide additional confidence that PA-dPEG24 can inhibit immune complex-initiated complement activation in human sera.

**Figure 3:**
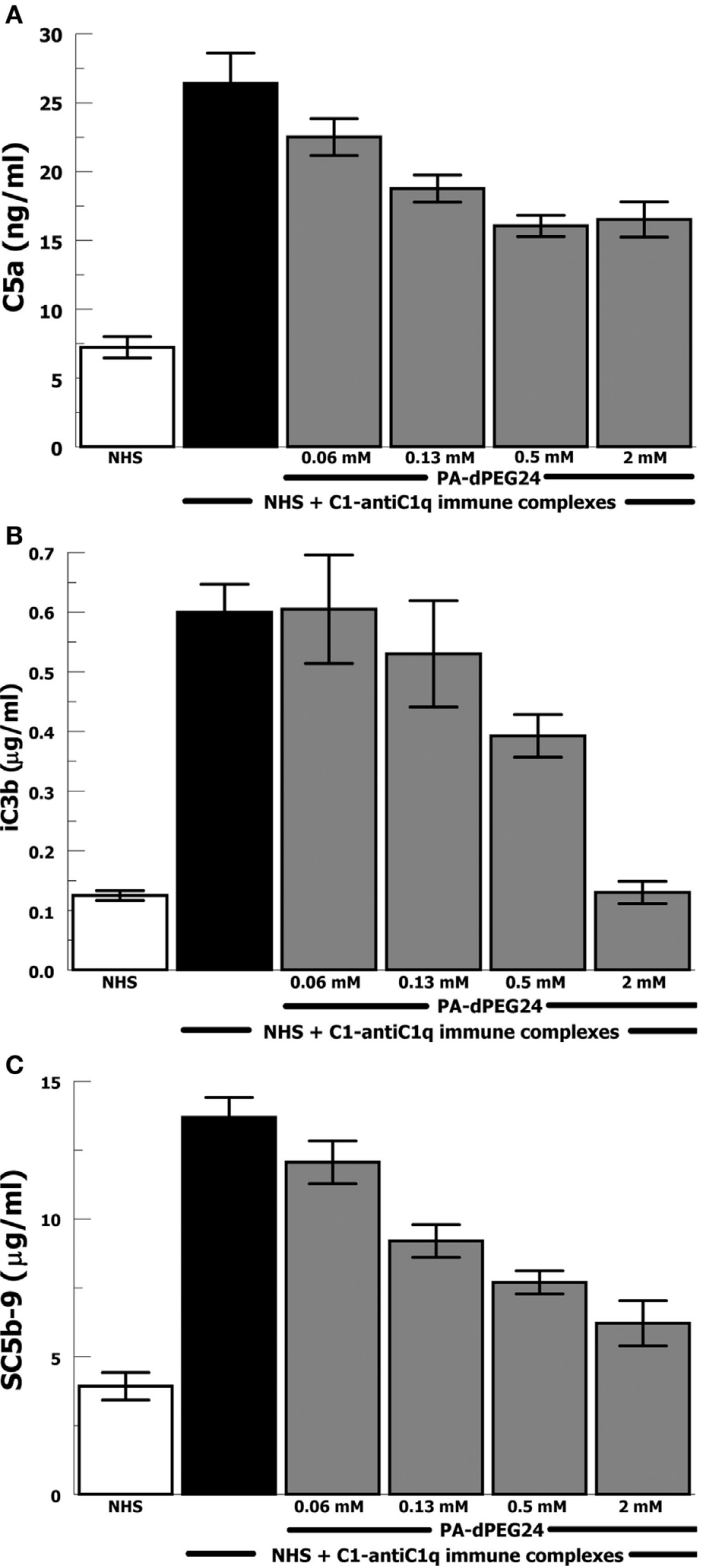
PA-dPEG24 inhibition of C1-anti-C1q immune complex-initated complement activation assayed by complement effectors. **(A)** PA-dPEG24 inhibition of C5a generation in normal human sera (NHS) stimulated with C1-anti-C1q immune complexes (IC). Data are means of (*n* = 4) independent experiments ± SEM. **(B)** PA-dPEG24 inhibition of iC3b generation in NHS stimulated with C1-anti-C1q IC. Data are means of (*n* = 4) independent experiments ± SEM. **(C)** PA-dPEG24 inhibition of SC5b-9 generation in NHS stimulated with C1-anti-C1q IC. Data are means of (*n* = 4) independent experiments ± SEM.

Due to the importance of anti-C1q antibodies in a subset of patients with SLE, we generated IC with human C1 and anti-C1q antibodies (goat). These IC activated NHS leading to robust generation of C5a, iC3b, and SC5b-9 (Figures 3A–C). PA-dPEG24 dose-dependently inhibited C1-anti-C1q generation of C5a in NHS at each concentration (*p* ≤ 0.03). C1-anti-C1q generation of iC3b was inhibited by 2 mM PA-dPEG24 (*p* < 0.02) to a level similar to NHS baseline. PA-dPEG24 dose-dependently inhibited C1-anti-C1q generation of SC5b-9 for concentrations ≥ 0.13 mM (*p* < 0.03). These results show that PA-dPEG24 can inhibit C1-anti-C1q immune complex-initiated complement activation in human sera.

### PA-dPEG24 Inhibition of PMA-Initiated NET Formation by Human Neutrophils

**Figure 4:**
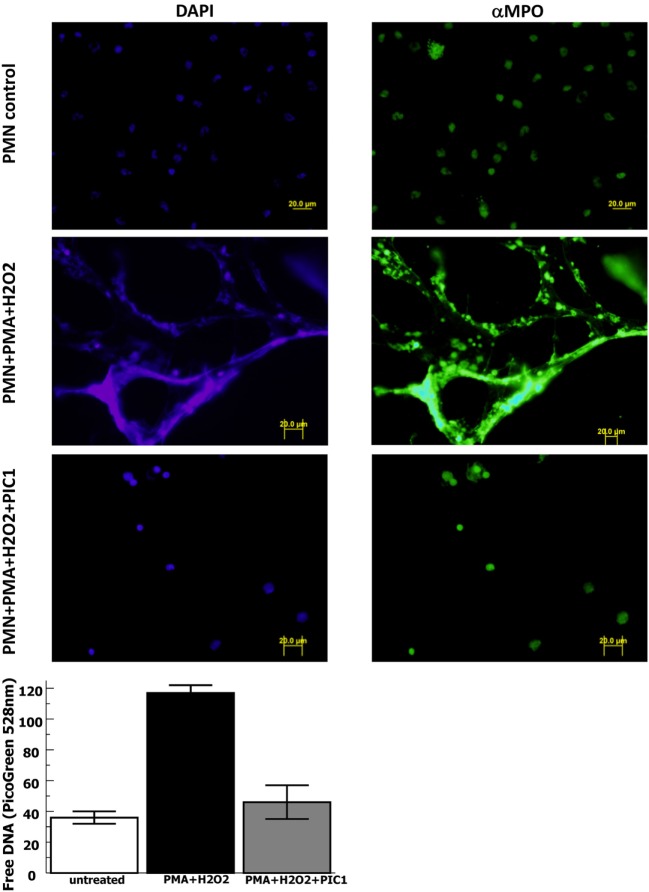
PA-dPEG24 inhibition of PMA-initiated neutrophil extracellular trap (NET) formation with human neutrophils (PMN) assayed by fluorescence microscopy and PicoGreen quantitation of free DNA. The first row shows unstimulated neutrophils, the second row shows neutrophils stimulated with PMA and hydrogen peroxide (H_2_O_2_), and third row shows neutrophils stimulated with PMA + H_2_O_2_ in the presence of 5 mM PA-dPEG24 peptide inhibitor of complement C1. The first columns are slides probed with DAPI to visualize DNA and the second column are slides probed with anti-myeloperoxidase antibody. The graph shows PA-dPEG24 (5 mM) inhibition of NET generation by human neutrophils stimulated with PMA + H_2_O_2_ assayed by PicoGreen. Data are means of (*n* = 3) independent experiments ± SEM.

**Figure 5:**
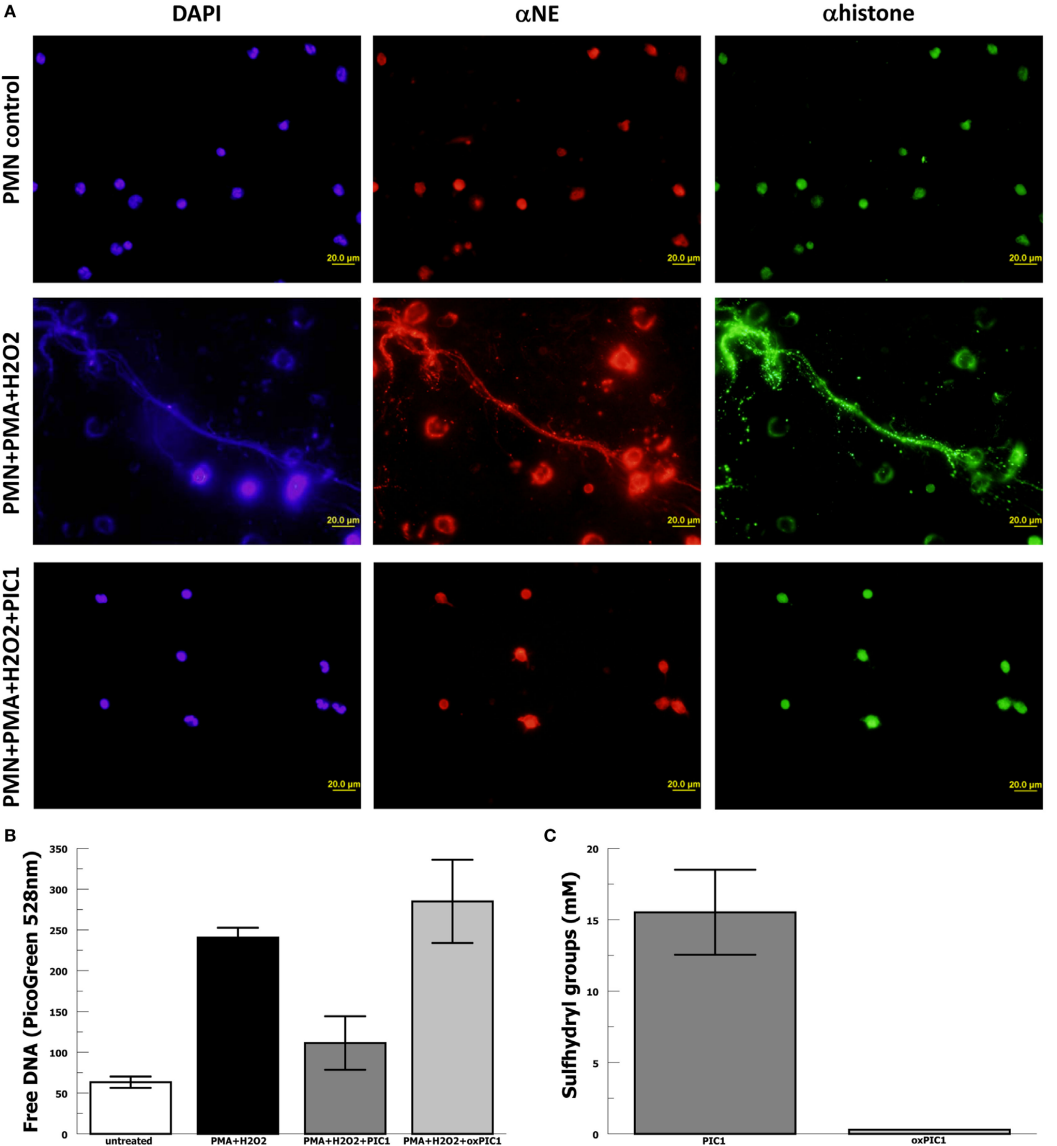
PA-dPEG24 inhibition of PMA-initiated neutrophil extracellular trap (NET) formation with human neutrophils (PMN). **(A)** PA-dPEG24 inhibition of PMA-initiated NET formation assayed by fluorescence microscopy for DNA (DAPI), neutrophil elastase (αNE), and histone H3 (αhistone). The first row shows unstimulated neutrophils, the second row shows neutrophils stimulated with PMA and hydrogen peroxide (H_2_O_2_), and third row shows neutrophils stimulated with PMA + H_2_O_2_ in the presence of 5 mM PA-dPEG24 peptide inhibitor of complement C1. The first columns are slides probed with DAPI to visualize DNA, the second columns are slides probed with anti-αNE antibody, and the third row is probed with anti-histone H3 antibody. Representative images are shown. **(B)** Oxidized PA-dPEG24 [ox peptide inhibitor of complement C1 (PIC1)] demonstrates no activity in the PMA-initiated NET assay by measuring free DNA with PicoGreen. Data are means of (*n* = 3) independent experiments ± SEM. **(C)** Oxidation of the sulfhydryl groups of PA-dPEG24 (oxPIC1) demonstrated with Ellman’s reagent. Data are means of (*n* = 3) independent experiments ± SEM.

In order to evaluate whether PA-dPEG24 can inhibit NET formation, we used purified human neutrophils and the commonly utilized stimulus phorbol 12-mystate 13-acetate (PMA) similar to methods described by Akong-Moore et al. (19). Two major components of NETs are extracellular DNA and myeloperoxidase which were visualized with DNA and anti-MPO antibody, respectively. Human neutrophils stimulated with PMA and H_2_O_2_ generated many NETs visualized by fluorescence microscopy, demonstrating extracellular DNA and extracellular MPO (Figure 4). In the presence of 5 mM PA-dPEG24, PMA and H_2_O_2_ did not generate NETs that could be identified by fluorescence microscopy. We then quantified NET formation by measuring free DNA in a PicoGreen-based assay from supernatants of human neutrophils stimulated in microtiter plate wells. PA-dPEG24 (5 mM) was able to inhibit free DNA elaboration by 2.6-fold (*p* = 0.01) in the presence of PMA and H_2_O_2_ compared with no inhibitor (Figure 4). This reduction for PA-dPEG24 was to a level similar to baseline without PMA. We also performed fluorescence microscopy utilizing the same experimental conditions, but probing for additional NET constituents extracellular neutrophil elastase and histone H3 (Figure 5A). Evaluating for these NET components also demonstrated that stimulation with PMA and H_2_O_2_ resulted in copious NET formation, which was inhibited in the presence of PA-dPEG24 (5 mM). In order to determine if the sulfhydryl groups in PA-dPEG24 were critical for inhibition of NET formation, we oxidized PA-dPEG24. The oxidized form of PIC1 demonstrated significantly less inhibition of PMA-initiated NET formation compared with unoxidized PA-dPEG24 (*P* = 0.006) and was not statistically different from the no PA-dPEG24 condition (*P* = 0.18) (Figure 5B). Complete oxidation of PA-dPEG24 sulfhydryl groups was verified using Ellman’s reagent which reacts with reduced sulfhydryl groups producing a color change that can be detected at 412 nm (Figure 5C). These results suggest that PA-dPEG24 can inhibit PMA-stimulated NET formation by human neutrophils.

### PA-dPEG24 Inhibition of MPO-Initiated NET Formation by Human Neutrophils

**Figure 6:**
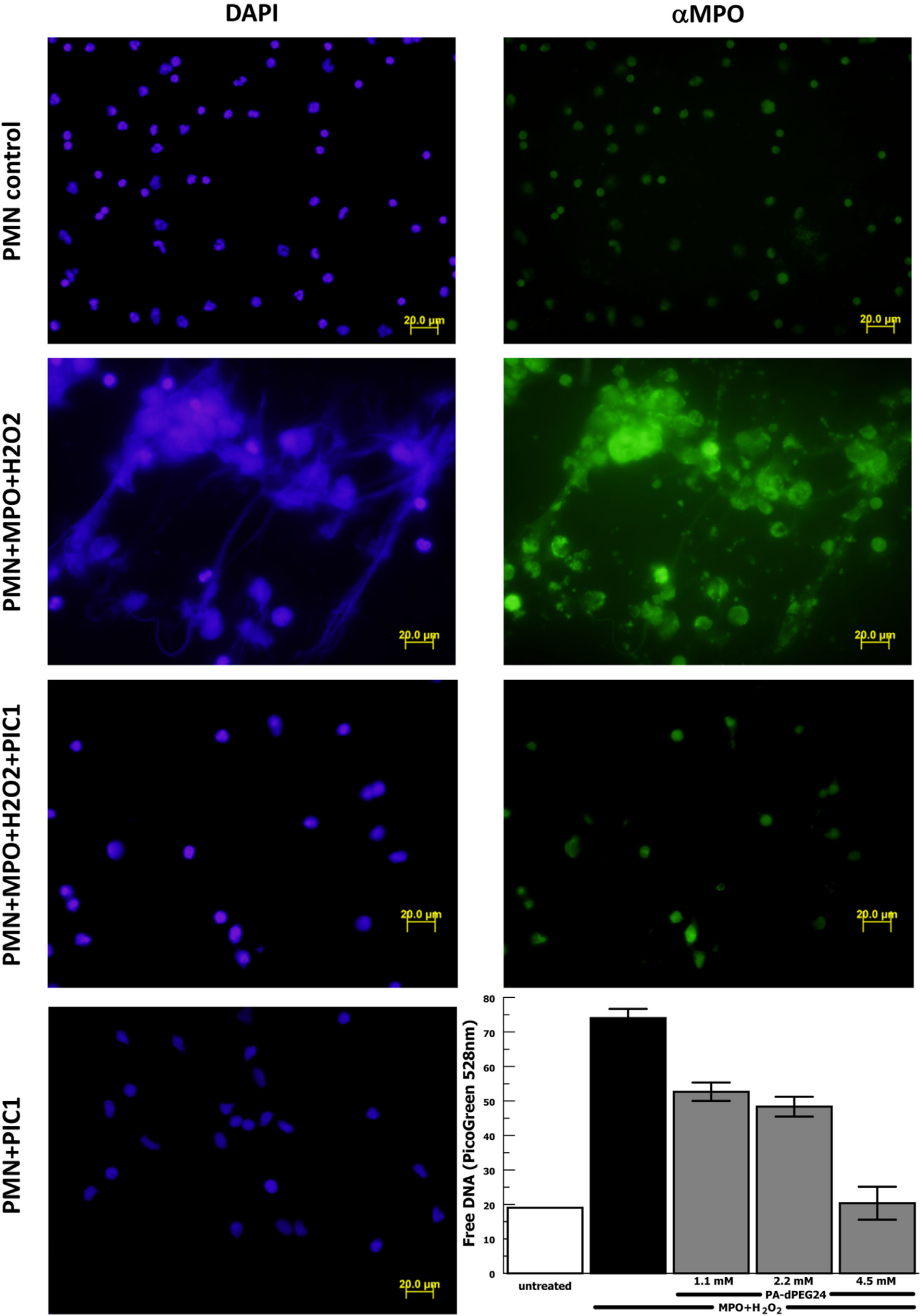
PA-dPEG24 inhibition of myeloperoxidase (MPO)-initiated neutrophil extracellular trap (NET) formation with human neutrophils (PMN) assayed by fluorescence microscopy and PicoGreen quantitation of free DNA. The first row shows unstimulated neutrophils, the second row shows neutrophils stimulated with MPO and hydrogen peroxide (H_2_O_2_), the third row shows neutrophils stimulated with MPO + H_2_O_2_ in the presence of PA-dPEG24 [peptide inhibitor of complement C1 (PIC1)], and the fourth column shows neutrophils incubated with PA-dPEG24 (PIC1) only. The first column shows slides probed with DAPI to visualize DNA. The second column shows slides probed with anti-MPO antibody. The graph shows PA-dPEG24 inhibition of NET generation by human neutrophils stimulated with MPO + H_2_O_2_ assayed by PicoGreen. Data are means of (*n* = 3) independent experiments ± SEM.

The results reported by Akong-Moore et al. (19) suggest that MPO is the critical mediator in PMA-stimulated NET formation; however, this was never tested using purified MPO. Thus, we repeated the above experiments with purified human neutrophils substituting purified MPO for PMA as the stimulus for NET formation. Neutrophil stimulation with purified MPO and H_2_O_2_ caused extensive NET formation visualized by DAPI staining and anti-MPO staining (Figure 6). NET formation in the presence of MPO and H_2_O_2_ was blocked with PA-dPEG24 (5 mM). Neutrophils incubated with PA-dPEG24 alone appeared normal by fluorescence microscopy. When NET formation was quantified by PicoGreen measurement, 1.1 mM of PA-dPEG24 lead to a 30% (*p* = 0.02) reduction in free DNA and 4.5 mM of PA-dPEG24 resulted in a 3.7-fold (*p* = 0.001) reduction in free DNA compared with stimulation with MPO, but no inhibitor (Figure 6). In the presence of MPO plus 4.5 mM PA-dPEG24, measured free DNA was not statistically different from unstimulated neutrophils. These results suggest that PA-dPEG24 inhibits NET formation *via* the MPO-mediated pathway.

### PA-dPEG24 Inhibition of Immune Complex-Initiated NET Formation by Human Neutrophils

**Figure 7:**
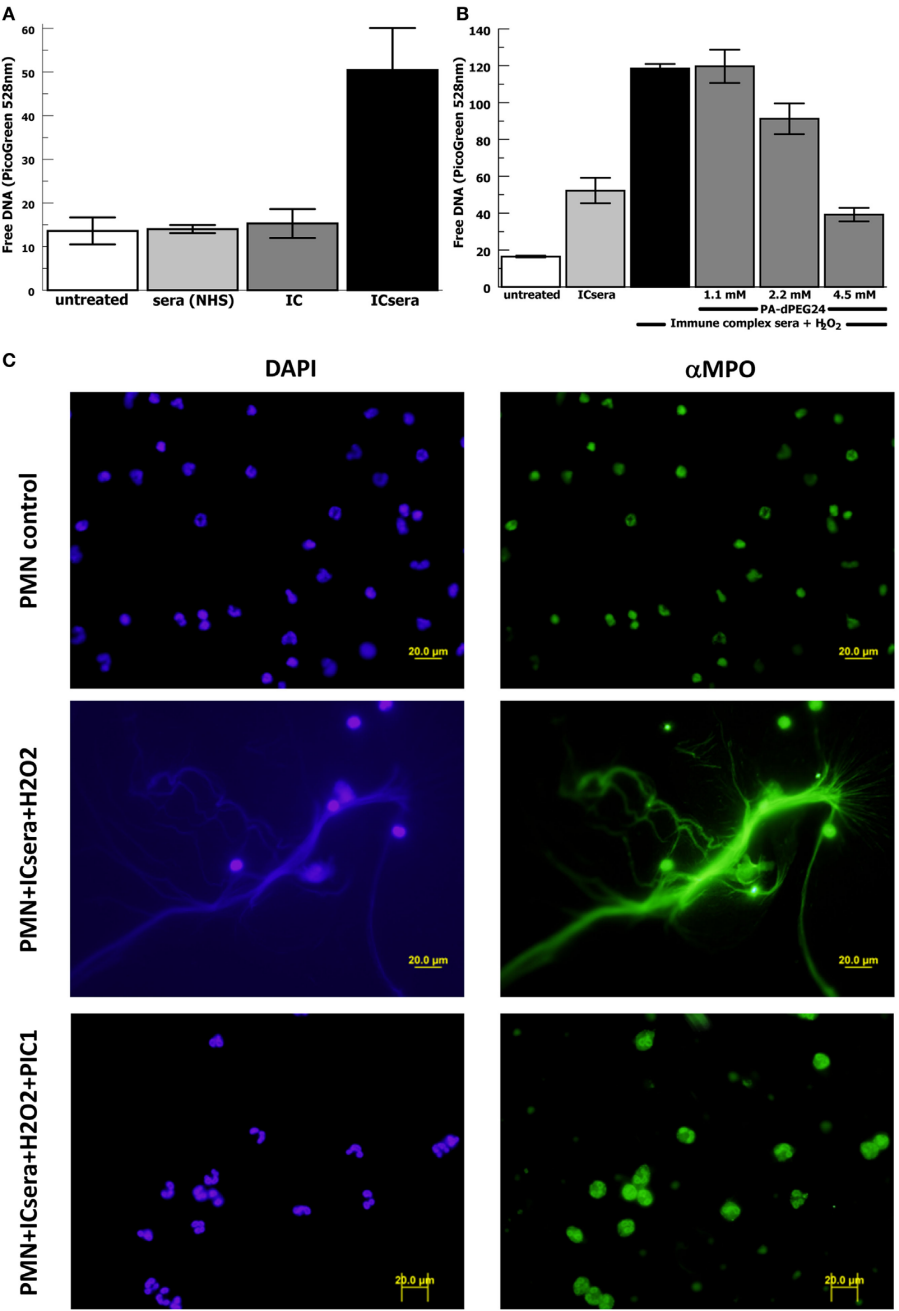
PA-dPEG24 inhibition of immune complex-activated serum-initiated neutrophil extracellular trap (NET) formation with human neutrophils (PMN) assayed by fluorescence microscopy and PicoGreen quantitation of free DNA. Ovalbumin–antiovalbumin immune complexes (IC) were utilized. **(A)** NET formation induced by PMNs incubated alone (untreated), with normal human sera (NHS), IC alone (IC), or immune-complex-activated human sera (ICsera). Data are means of (*n* = 5) independent experiments ± SEM. **(B)** PA-dPEG24 inhibition of NET generation by human neutrophils stimulated with ICsera assayed by PicoGreen. Data are means of (*n* = 4) independent experiments ± SEM. **(C)** The first row shows unstimulated neutrophils, the second row shows neutrophils stimulated with ICsera and hydrogen peroxide (H_2_O_2_), and the third row shows neutrophils stimulated with ICsera + H_2_O_2_ in the presence of PA-dPEG24 (peptide inhibitor of complement C1). The first column is slides probed with DAPI to visualize DNA and the second column is slides probed with anti-MPO antibody.

Next we evaluated for a potential relationship between immune complex-initiated complement-activated human sera and NET formation by human neutrophils, because this has not been previously explored. We activated complement in normal human sera with ovalbumin–antiovalbumin IC as was performed in Figure 2. The immune complex-initiated complement-activated human sera were then incubated with purified human neutrophils resulting in NET formation quantified by free DNA measurement with PicoGreen. We initially evaluated the relative contribution of IC by them compared with immune complex-activated human sera for generation of NETs. The presence of IC by themselves did not significantly (*p* = 0.39) increase NET formation compared with neutrophils alone (Figure 7A). However, immune complex activation of complement in sera increased NET formation >20-fold (*p* = 0.009) compared with IC alone after subtracting the background. These results suggest that immune complex-initiated complement-activated sera are a strong stimulus for NET formation.

We then evaluated whether H_2_O_2_ further enhanced NET formation by immune-complex-activated human sera (ICsera) and found that it approximately doubled the signal (Figure 7B). Testing of PA-dPEG24 inhibition of NETosis was performed with PA-dPEG24 being added after complement activation of the sera by IC that had already been allowed to occur; therefore, any effect of PA-dPEG24 on NETosis happened downstream of complement activation. In the presence immune complex-initiated complement-activated sera and H_2_O_2_, 2.2 mM PA-dPEG24 decreased free DNA by 23% (*p* = 0.037) and 4.5 mM PA-dPEG24 decreased free DNA by threefold (*p* < 0.001) compared with no inhibitor (Figure 7B). These conditions were also visualized by fluorescence microscopy (Figure 7C). In the presence of PA-dPEG24 (5 mM) and ICsera, no NETs were identified by fluorescence microscopy. These findings demonstrate immune complex-initiated complement-activated human sera can stimulate human neutrophils to form NETs and that this can be inhibited with PA-dPEG24. Taken together, these experiments consistently show that PA-dPEG24 can inhibit NET formation by human neutrophils initiated by various stimulants.

## Discussion

The experiments shown above demonstrate that PA-dPEG24 can inhibit immune complex-initiated complement activation and the generation of pro-inflammatory complement effectors. This suggests that complement inhibitory peptides could potentially moderate aspects of pathogenesis in diseases where immune complex activation of the complement system plays a vital role. Additionally, we utilized C1 and anti-C1q antibodies as a novel immune complex modeling a type of immune complex that could be predicted to be formed in the plasma of SLE patients with circulating anti-C1q antibodies. PA-dPEG24 also blocked complement activation by C1-anti-C1q IC, consistent with the other immune complex types tested.

We have previously shown that PA-dPEG24 inhibits hemolysis of antibody-sensitized erythrocytes *in vitro* and *in vivo* (25, 26). The studies described here expand upon these observations demonstrating that PA-dPEG24 can inhibit immune complex-initiated complement activation. Thus, there is consistency in PA-dPEG24 inhibition of the classical complement pathway for various stimuli.

Our NETosis experiments demonstrate that immune complex-initiated complement-activated human sera can initiate NET formation. This adds to prior observations that IC by themselves can initiate NET formation *via* Fc receptors (14–17). Under the experimental conditions we used, the contribution of immune complex-initiated complement activation to NET formation was much greater than that of IC alone. Given the presence of IC in several rheumatologic diseases, including active SLE disease, it is reasonable to hypothesize that immune complex-initiated complement activation may contribute to NET formation in SLE and potentially other rheumatologic diseases.

These experiments also show that PA-dPEG24 can inhibit NET formation by human neutrophils initiated by PMA, MPO, or immune complex-activated human sera. Given that PMA has been shown to stimulate NET formation *via* an MPO-mediated pathway (19) and our results utilizing purified MPO, it is likely that PA-dPEG24 blocks NET formation by inhibiting MPO (27). The ability of PA-dPEG24 to inhibit NET formation after immune complex-initiated complement activation of human sera has occurred, suggests that this mechanism of NET formation may also occur *via* an MPO-mediated pathway. This adds immune complex-initiated complement activation as a new stimulus for MPO-mediated NET formation to others described previously (19–21).

Together, these data show that the PA-dPEG24 peptide can block both immune complex-initiated complement activation and inhibit NETosis. The vital role of NETs in autoimmune diseases was nicely reviewed by Brinkmann and Zychlinsky (37) describing the role of NETs in SLE noting the potential role of increased C1q deposition inhibiting DNaseI and preventing NET degradation. Given that the peptide core of PA-dPEG24 strongly binds C1q (25), warrants future studies to evaluate a potential effect on NET degradation. A more recent review of NETosis contribution to autoimmune diseases written by Gupta and Kaplan (38) details the role of NETs in SLE, ANCA-associated vasculitis, rheumatoid arthritis, antiphospholipid antibody syndrome, type 1 diabetes, and renal inflammatory diseases. This provides proof of concept that peptides can potentially be developed to inhibit these two important contributors to rheumatologic pathology that is currently untargeted by available therapies. The next steps for evaluation of PA-dPEG24 will be testing in animal models of immune complex-mediated diseases and animal models of NET-mediated diseases.

## Data Availabilty

The raw data supporting the conclusions of this manuscript will be made available by the authors, without undue reservation, to any qualified researcher.

## Ethics Statement

Blood from healthy donors was obtained with written informed consent under an Eastern Virginia Medical School IRB approved protocol, 02-06-EX 0216.

## Author Contributions

PH, NK, and KC provided the concepts for the study, designed the tests, and wrote the manuscript. PH and AE performed the assays and data collection. PH and KC performed the analysis and statistics. All authors reviewed the manuscript for content, provided suggestions, and approved the final manuscript.

## Conflict of Interest Statement

The authors declare that the research was conducted in the absence of any commercial or financial relationships that could be construed as a potential conflict of interest.

## Abbreviations

NET, neutrophil extracellular trap; MPO, myeloperoxidase; PIC1, peptide inhibitor of complement C1; PA-dPEG24, IALILEPICCQERAA-dPEG24; SLE, systemic lupus erythematosus.
